# Oral impacts on daily performances and its socio-demographic and clinical distribution: a cross-sectional study of adolescents living in Maasai population areas, Tanzania

**DOI:** 10.1186/s12955-020-01444-7

**Published:** 2020-06-12

**Authors:** Lutango D. Simangwa, Ann-Katrin Johansson, Anders Johansson, Irene K. Minja, Anne N. Åstrøm

**Affiliations:** 1 Department of Clinical Dentistry - Cariology, Faculty of Medicine, University of Bergen, Bergen, Norway; 2 Department of Clinical Dentistry - Prosthodontics, Faculty of Medicine, University of Bergen, Bergen, Norway; 3 Department of Restorative Dentistry, School of Dentistry, Muhimbili University of Health and Allied Sciences, Dar Es Salaam, Tanzania; 4 Department of Clinical Dentistry – Community Dentistry, Faculty of Medicine, University of Bergen, Bergen, Norway

**Keywords:** Adolescents, Maasai population areas, Oral diseases, Oral impacts on daily performance, And sociodemographic factors

## Abstract

**Background:**

In a global perspective, oral health among adolescents has improved during recent decades. However, oral problems still persist especially in many underprivileged societies. This study aimed to estimate the prevalence of oral impacts in adolescents and to identify important clinical- and socio-demographic covariates. In addition, this study compares Maasai and non-Maasai adolescents regarding any association of socio-demographic and clinical covariates with oral impacts on daily performances.

**Methods:**

A total of 989 adolescents were invited from 23 randomly selected public primary schools in Monduli and Longido districts, Tanzania. All adolescents attending 6th grade classes were invited to participate. A total of 930 accepted and of those 24 were excluded, leaving 906 (91.6%) participants for the study.

**Results:**

A total of 143/906 (15.8%) had at-least one oral impact on daily performances (OIDP > 0), 14.6% among the Maasai and 20.5% among the non-Maasai ethnic group. Cluster adjusted logistic regression revealed that: adolescents from Longido district (OR = 0.4) and adolescents with good oral hygiene (OR = 0.7) were less likely to report OIDP> 0 and; non Maasai (OR = 1.6), those with least poor parents (OR = 2.0), DMFT> 0 (OR = 3.1) and those with positive answers to questions regarding TMD pain, 2Q/TMD > 0 (OR = 3.9) were more likely to report OIDP> 0. Stratified logistic regression by ethnicity revealed that, among the non-Maasais, older adolescents (OR = 3.7, 95% CI 1.1–12.8), those with DMFT> 0 (OR = 3.3 (1.2–9.0) and 2Q/TMD > 0 (OR = 9.0, 95% CI 3.3–25.0) were more likely to report at least one OIDP. The corresponding figures among the Maasais were (OR = 0.9, 95% CI 0.5–1.7), (OR = 2.8, 95% CI 1.4–5.5) and (OR = 3.0, 95% CI 1.7–5.2), respectively.

**Conclusions:**

The prevalence of oral impacts was moderate but higher among the non-Maasai- than Maasai-adolescents attending rural primary schools in the Maasai population areas of Tanzania. This study also confirmed socioeconomic and oral clinical disparities in OIDP, some of which differed according to ethnicity. Caries experience and self-reported TMD pain associated more strongly with OIDP among the non-Maasais than among the Maasais. These results are important for public oral health decision makers who plan strategies for optimal primary oral health care and quality of life among adolescents belonging to minority groups in Tanzania.

## Background

In a global perspective, oral health among adolescents has improved during recent decades. However, oral diseases like dental caries and erosion as well as dental fluorosis and periodontal diseases still prevail in certain groups and particularly so in underprivileged societies [1–3]. Studies have confirmed that oral diseases have a negative impact on the quality of life and well-being of adolescents [4–6]. Evidence suggests a gap between professional and subjective evaluations of oral health, possible affecting for example treatment need [7].

Recognizing the growing importance of quality of life measures in health care, oral health related quality of life (OHRQoL) inventories are available to address functional, psychological and social consequences of oral diseases and also to complement clinical measures and evaluate treatment outcomes [8–10]. Adulyanon et al. [11] developed the Oral impact on daily performance (OIDP) inventory, one of the most commonly used OHRQoL instruments, to be used either as a generic or a disease specific measure of oral health related quality of life. The OIDP inventory is based on the conceptual framework of the World Health Organization’s International Classification of Impairments, Disabilities and Handicaps, ICIDH [12], and has been amended for use in dentistry by Locker [13]. This inventory covers the ultimate disability and handicap dimensions in the ICIDH model and includes 8 items assessing physical, psychological, and social dimensions of daily living [11]. Two versions of the OIDP inventory have been applied in the literature, one for adults and another one for children and adolescents. The Child OIDP was developed to fit children’s cognitive stage of development and was initially tested among 11–12 year old school children in Thailand [14]. This inventory has been widely applied across low and high-income countries [15–17] and has shown acceptable psychometric properties when applied to adolescent populations globally [6, 15, 18].

However, its application in minority groups, such as the Maasai, is scarce although indigenous populations around the world experience disproportionate burden of oral diseases and conditions [16]. Moreover, the clinical and socio-demographic distribution of OIDP have not been investigated to the same extent in adolescents as in adults [17]. Thus, information of the performance of OHRQoL instruments across socio-cultural minority groups within and across countries has been requested [6].

The Maasai is a unique and popular tribe due to their long preserved culture. Despite education, civilization and western cultural influences, the Maasai people have clung to their traditional way of life [19]. In Tanzania, The Maasais are considered socially disadvantaged because the Maasai reside in a semi-arid ecology prone to erratic rainfall and periodic drought [20]. Such vulnerability may lead to high food insecurity and poor health outcomes [21]. In addition, the Maasai communities tend to live in remote rural areas and also have a relatively poor command of Swahili, the national language of Tanzania. These factors decrease opportunities for obtaining good health services and educational attainment and might thus affect their well-being and quality of life [22].

To the best of our knowledge, no retrievable data/information considers evaluation of OHRQoL in adolescents living in Maasai populated areas of Tanzania. Focusing on Maasai and non-Maasai adolescents and using the Child OIDP inventory, this study aimed to estimate the prevalence of oral impacts and to identify important clinical- and socio-demographic covariates. In addition, this study compares Maasai and non-Maasai adolescents regarding any association of socio-demographic and clinical covariates with oral impacts on daily performances.

## Methods

### Study design and participants

A cross-sectional study was conducted among 12–14-year-old adolescents in Maasai populated areas of Monduli and Longido districts, in the Arusha region, Tanzania, from June to November 2016. A list of all primary schools was obtained from both districts (including 100 schools). Urban and private schools were excluded due to the reason that the majority of Maasais live in rural remote areas and are relatively poor and therefore won’t be able to bring their children in private schools which are relatively expensive. The inclusion criteria were therefore, the adolescents expected to be in age ranging from 12 to 14 year old attending rural public primary schools of Monduli and Longido districts. The exclusion criteria were adolescents attending urban and private primary schools, those who were absent during the interview/oral examination day and those with difficulties in learning. After excluding urban and private schools, 23 (13 from Monduli and 10 from Longido) from a total of 66 (38 from Monduli and 28 from Longido), eligible rural public primary schools were randomly selected using a one-stage cluster sample design with school as the primary sampling unit. A class expected to contain adolescents aged 12–14 years was identified (6th grade) in each selected school. All adolescents available in the identified classes were invited to participate. The sample size was estimated based on the assumption that the prevalence of dental erosion among adolescents was 50%. The estimated minimum sample size for this study, 845 adolescents was obtained by assuming a margin error of 5% and, confidence intervals of 95%. Details about the sampling technique have been described elsewhere [23]. Thus, in this study, we did a secondary analysis of a study planned for other purposes. Briefly, we invited a total of 989 adolescents to participate in the study and 930 of them accepted this invitation. The final sample included 906 adolescents after having excluded 24 due to too low or high age, giving a response rate of 91.6%.

### Interview and questionnaires

Closed- and open-ended questions were used to assess information in face- to- face interviews. The interview schedule was constructed in English, translated into Swahili and back-translated to English independently by qualified translators from the University of Dar Es Salaam, Tanzania. A pilot test was performed with a group of 50 adolescents aged 12–14 years who were not included in the main study. Two trained medical nurses performed face-to-face interviews in Swahili/Maa (Maasai language) in a school setting. Each adolescent was interviewed separately, inside a classroom or outside the classroom (under a tree), in order to achieve privacy.

Socio-demographic factors were assessed in terms of age, ethnicity, sex, place of residence, mother’s education, household socio-economic status (perceived affluence of my household) and household wealth index [24]. Ethnicity was assessed by asking “what is your ethnic group?” During analysis, the response ethnical categories 1 = Maasai, 2 = Meru, 3 = Arusha and 4 = others were dichotomized to 1 = Maasai (from option 1) and 2 = non-Maasai (from option 2, 3 and 4). Parents’ education was assessed by asking *what is the highest level of school your mother/father has attended?* Response categories were (0) none, (1) she/he started but did not complete primary school, (2) completed primary school (3) she/he started but did not complete secondary school, (4) she/he completed secondary school, (5) she/he started but did not complete college/university, (6) completed college/university, (7) I don’t know. For analyses, the options were dichotomized as (0) for low education (from options 0, 1, 2, 3 and 7) and (1) for high education (from options 4, 5 and 6). The wealth index was constructed by assessing the presence of durable household assets which indicate family wealth (i.e. radio, television, refrigerator, mobile telephone, cupboard, bicycle and motorcycle). These were recorded as (Yes) “available and in working condition” or (No) “not available and/or not in working condition” and then the principle component analysis (PCA) method was used to construct a wealth index [24]. Using the first component, we categorized the wealth index into 1st quartile, 2nd quartile, 3rd quartile and 4th quartile implying the poorest, poorer, less poor and least poor, respectively. The wealth index is a measure of a household’s cumulative living standard, usually calculated by summing up data on a household’s ownership of selected assets. PCA works best when the household asset variables are correlated to each other and when the distribution of variables varies across the households. It is those assets that are more unequally distributed between households that are given more weight [25]. For example, assets owned by majority of households would exhibit no variation between households and would be zero weighted and thus of no use in differentiating the wealth of a particular family. Therefore in our study we excluded some of the assets eg animals they had due to the reason that they were owned by majority of communities.

Oral health related quality of life was measured using a Kiswahili (Tanzania national language) version of the eight item Child OIDP inventory, previously shown to be reliable and valid when applied to adolescents in Tanzania [6]. The Child OIDP frequency index referred to difficulty carrying out eight daily life activities *“During the past 3 months, how often have problems with your mouth or teeth caused you any difficulty with; eating and enjoying food, speaking and pronouncing clearly, cleaning teeth, sleeping and relaxing, smiling and laughing, emotional status, socialization and contact with people.* The original responses were (0) never, (1) once or more a month, (2) once or more a week and (3) every day/nearly every day. In the statistical analyses, the items were dichotomized as 0 not affected (comprising original responses 0) and 1 affected (comprising original responses 1, 2 and 3). A Child-OIDP simple count (SC) score (range 0–8) was constructed by summing the dichotomized frequency items of (0) not affected and (1) affected and subsequently dichotomized into 0 (no impacts) and 1 (at least one impact). In its original form, OIDP scores are calculated by multiplying frequency and severity scores of daily performances [26]. However, evidence suggests the use of either frequency or severity scores for reasons of simplicity and efficiency [26]. The internal consistency reliability (Cronbach’s alpha based on standardized items) for the child OIDP inventory was 0.82, which is in agreement with previous figures (0.84 to 0.85) from Tanzania [5, 27]. Validity of OIDP is further confirmed in this study by associations with clinical variables in the expected direction.

The two epidemiological questions regarding temporomandibular disorder pain (2Q/TMD), were: “*Do you have pain in your temple, face, jaw or jaw joint once a week or more?”* and *“Does it hurt once a week or more when you open your mouth or chew?”* The response was either “*yes”* or *“no”* and positive answer to any of the two questions was considered affirmative to self-reported TMD pain diagnosis [28]. Thus, a positive answer to one or both of the two epidemiological questions was recorded as 2Q/TMD > 0 and a negative answer to both epidemiological questions was recorded as 2Q/TMD = 0.

### Oral clinical examination

All clinical examinations were performed by the first author (LS) under natural day light with the adolescent sitting on a chair. The teeth were cleaned and dried by sterile gauze and isolated by cotton rolls. Disposable mouth mirrors and probes were used. The examiner was trained and calibrated at the Department of Clinical Dentistry, University of Bergen, Norway.

Details considering the clinical oral examination have been described elsewhere [23]. Oral hygiene was assessed using the Simplified Oral Hygiene Index (OHI-S) [29]. Plaque and calculus was assessed on teeth (16, 11, 26, 36, 31 and 46). The scores were (0) for no plaque/calculus present, (1) for plaque or supra-gingival calculus covering not more than one third of the tooth surface, (2) for plaque or supra-gingival calculus covering more than one third but less than two thirds of the tooth surface, and (3) for plaque or supra-gingival calculus covering more than two thirds of the tooth surface. For each individual, the plaque and calculus scores were summed up and divided by total number of teeth examined to obtain the Simplified Debris Index (DI-S) and simplified calculus index (CI-S). The OHI-S was constructed by summing up the DI-S and CI-S. During analysis the OHI-S scores were dichotomized into 1 = good oral hygiene (OHI-S < 1) and 2 = poor oral hygiene (OHI-S ≥ 1). Gingival health was assessed by Gingival Bleeding Index (GBI) and was recorded as positive or negative following a gentle probing on gingival sulcus of the index teeth after 10 s [30]. Dental caries was assessed according to criteria specified by WHO [31] and was recorded as 0-Sound, 1-Decayed, 2-Filled, with decay, 3-Filled, no decay, 4-Missing due to caries, 5-Missing for other reason, 6-Fissure sealant, 7-Bridge abutment, special crown or veneer/implant, 8-Unerupted tooth (crown)/unexposed root, 9-Not recorded. Dental fluorosis was assessed by Thylstrup- Fejerskov - index (TF-index) [32] and was recorded as score 0 to 9 score with score 0 – not affected and score 1–9 showing varying degree of dental fluorosis. Dental erosion on palatal and facial surfaces of maxillary anterior teeth was recorded according to Johansson et al. [33], recording as score 0 to 4, with score 0 not affected by erosion and score 1–4 affected by dental erosion at different levels. Grading of first molar cuppings by Hasselkvist et al. [34], recorded as score 0 to 4, with score 0 no cupping and score 1–4 varying degree of cuppings. Tooth wear was graded as a full mouth recording of occlusal/incisal surfaces according to Carlsson et al. [35], recorded as score 0 to 4, with score 0 no tooth wear and score 1–4 varying degree of tooth wear.

The inter-examiner (between LS and AKJ) Cohen’s Kappa for dental erosion in all examined teeth surfaces was 82.4%. Duplicate clinical examinations (intra-examiner concordance), 3 weeks apart, including 93 randomly selected participants, gave a Kappa value for caries experience (DMFT> 0), dental fluorosis (TF-index) and dental erosion of 98.3, 86.8 and 69.4, respectively.

### Statistical analysis

The Statistical Package for Social Sciences (SPSS) for PC version 24 (IBM corporation, Armonk, NY, USA) was used to analyze the data. STATA 14.2 (Stata corporation, Lakeway drive college station, Texas, USA) was used to adjust for cluster effect of school. Bivariate analysis was performed by cross tabulations and Pearson’s chi-square statistical test. Inter- and intra-examiner concordances were determined using percentage agreement and Cohen’s Kappa. Internal consistency reliability was assessed using Cronbach’s alpha. Stepwise multiple variable logistic regression analysis (Odds Ratio and 95% CI) was conducted with OIDP regressed on sociodemographic and oral clinical variables that were statistically significantly associated with OIDP in crude (unadjusted) analysis. In each step Nagelkerke’s R^2^ was calculated which denotes a pseudo R square that generalize the coefficient of determination with values between 0 and 1 where 0 denotes that the model do not explain anything of the variation- whereas 1 denotes a model explaining all variation in the dependent variable. The effect of two –way interaction terms between ethnic group and socio-demographic- and clinical variables on OIDP was tested to explore whether any association of socio-demographic- and clinical variables with OIDP differed between the ethnic groups.

## Results

### Sample profile

A total of 906 (56.1% females), 12–17-year-old (mean age 13.4 years SD 1.2) adolescents attending grade 6 were interviewed and underwent an oral clinical examination at school. Out of these 906, 479 (52.9%) were from Monduli district, 721 (79.6%) belonged to the Maasai- and 185 (20.4%) to the non-Maasai ethnic groups. Table 1 depicts the percentage distribution of study participants’ sociodemographic- and clinical characteristics in the pooled sample and according to Maasai and non-Maasai ethnicity. A majority (91.2%) of the adolescents were dental caries free (DMFT = 0) and 48.6% of the adolescents had severe dental fluorosis (TF score 5–9). A total of 11.8% of the adolescents self-reported Temporomandibular Disorder pain (2Q/TMD > 0). Most socio-demographic and clinical characteristics differed statistically significantly by ethnic group (Table 1).

**Table 1:** Frequency distribution of sociodemographic and clinical characteristics in a total sample (*n* = 906) and by ethnicity

Variable	Total % (n)	Maasai % (n)	Non-Maasai % (n)	*P-value
**District of residence**				
Monduli	52.9 (479)	58.0 (418)	33.0 (61)	
Longido	47.1 (427)	42.0 (303)	67.0 (124)	< 0.001
**Sex**				
Male	43.9 (398)	43.1 (311)	47.0 (87)	
Female	56.1 (508)	56.9 (410)	53.0 (98)	0.341
**Age**				
12–14 years	87.3 (777)	86.5 (610)	90.3 (167)	
15–17 years	12.7 (113)	13.5 (95)	9.7 (18)	0.173
**Wealth index**				
Poorest	48.8 (438)	57.3 (408)	16.2 (30)	
Least poor	51.2 (459)	42.7 (304)	83.8 (155)	< 0.001
**Mother’s education**				
Low (≤ primary school)	861 (95.0)	96.8 (698)	88.1 (163)	
High (≥ secondary school)	45 (5.0)	3.2 (23)	11.9 (22)	< 0.001
**Oral hygiene status**				
Poor	65.6 (594)	68.5 (508)	54.1 (100)	
Good	34.4 (312)	31.5 (213)	45.9 (85)	< 0.001
**Gingival bleeding**				
No	59.1 (535)	55.6 (401)	72.4 (134)	
Yes	40.9 (371)	44.4 (320)	27.6 (51)	< 0.001
**DMFT**				
DMFT = 0	91.2 (826)	92.6 (668)	85.4 (158)	
DMFT > 0	8.8 (80)	7.4 (53)	14.6 (27)	0.002
**Dental fluorosis**				
TF score 0–4	51.4 (466)	47.9 (345)	65.4 (121)	
TF score 5–9	48.6 (440)	52.1 (376)	34.6 (64)	< 0.001
**Dental erosion**				
Grade 0	69.8 (632)	73.2 (528)	56.2 (104)	
Grade > 0	30.2 (274)	26.8 (193)	43.8 (81)	< 0.001
**Tooth wear**				
Grade 0	54.0 (489)	55.1 (397)	49.7 (92)	
Grade > 0	46.0 (417)	44.9 (324)	50.3 (93)	0.194
**TMD pain**				
2Q/TMD = 0	88.2 (799)	88.8 (640)	85.9 (159)	
2Q/TMD > 0	11.8 (107)	11.2 (81)	14.1 (26)	0.289

*Pearson’s Chi-square test

### OIDP by socio-demographic and oral clinical features

A total of 143/906 (15.8%) of adolescents investigated reported at-least one oral impact on daily performances (OIDP > 0). The frequency of the reported impacts was: eating and enjoying food (7.9%), speaking and pronouncing clearly (4.4%), cleaning teeth (10.5%), sleeping and relaxing (3.9%), smiling and laughing (2.0%), maintaining usual emotional state (2.1%), carrying major school work or social role (2.2%) and enjoying contact with people (2.1%) (not presented in table).

As depicted in Table 2, socio-demographic and clinical features in terms of district of residence, ethnicity and wealth index and oral hygiene status, DMFT (Decayed Missing Filled Teeth), dental fluorosis and self-reported TMD pain status associated statistically significantly with oral impacts (OIDP> 0). Oral impacts were more frequently reported by adolescents from Monduli districts than among those from Longido (21.9% versus 8.9%, *p* < 0.001). Oral impacts were most frequently reported among participants with poor oral hygiene status, caries experience (DMFT> 0) and self-reported TMD pain.

**Table 2:** Distribution of OIDP according to sociodemographic features and clinical indicators of oral diseases/problems

Variable	Categories	OIDP > 0% (n)	*P*-value*
District of residence	Monduli	21.9 (105)	
	Longido	8.9 (38)	< 0.001
Sex	Female	14.2 (72)	
	Male	17.8 (71)	0.133
Age	12–14 years	15.2 (118)	
	15–17 years	22.1 (25)	0.061
Ethnicity	Maasai	14.6 (105)	
	Non-Maasai	20.5 (38)	0.047
Wealth index	Poorest	11.9 (52)	
	Least poor	19.4 (89)	0.002
Mother’s education	Low (≤ primary school)	15.4 (133)	
	High (≥ secondary school)	22.2 (10)	0.224
Oral hygiene status	Poor	18.2 (108)	
	Good	11.2 (35)	0.006
Gingival bleeding	No	14.8 (79)	
	Yes	17.3 (64)	0.313
DMFT	DMFT = 0	13.7 (113)	
	DMFT > 0	37.3 (30)	< 0.001
Dental fluorosis	TF score 0–4	11.4 (53)	
	TF score 5–9	20.5 (90)	< 0.001
Dental erosion	Grade 0	16.8 (106)	
	Grade > 0	13.5 (37)	0.215
Tooth wear	Grade 0	14.9 (73)	
	Grade > 0	16.8 (70)	0.445
TMD pain	2Q/TMD = 0	12.3 (98)	
	2Q/TMD > 0	42.1 (45)	< 0.001

*Pearson’s Chi-square test

Table 3 depicts adjusted odds ratios (OR) and 95% CI for OIDP (Oral Impacts on Daily Performace) by sociodemographic features and oral diseases/problems. District of residence, sex, ethnicity and wealth index were entered in step 1, providing a model fit of Nagelkerke’s *R*^2^ = 0.103, Model Chi-Square = 55.407, df = 4 and *p* < 0.001. District, ethnicity, wealth index and age were statistically significantly associated with OIDP in the first step of the model. Entering oral hygiene status, DMFT> 0, dental fluorosis (TF 5–9) and 2Q/TMD > 0 in the second step improved model fit to Nagelkerke’s *R*^2^ = 0.199, model Chi-square = 110.178, df = 8 and *p* < 0.001. In the second step of the model, district (OR = 0.4, CI 0.3–0.7), ethnicity (OR = 1.6, CI 1.1–2.3), wealth index (OR = 2.0, CI 1.2–3.3), oral hygiene status (OR = 0.7, CI 0.5–0.9), DMFT > 0 (OR = 3.1, CI 2.1–4.5) and 2Q/TMD > 0 (OR = 3.9, CI 2.4–6.2) were significant covariates of OIDP> 0. Adjusting for cluster effect did not change the ORs, but there was a small widening of the confidence intervals, only.

**Table 3:** Logistic regression for the association between adolescents’ social and clinical characteristics and OIDP, odds ratios (OR) ad 95% confidence interval (CI)

Variable	Step 1 OR (95% CI)	Step 2 OR (95% CI)
**District of residence**		
Monduli	1	1
Longido	0.3 (0.2–0.4)^b^	0.4 (0.3–0.7)^b^
**Age**		
12–14 years	1	1
15–17 years	1.4 (1.0–2.1)	1.2 (0.9–1.8)
**Ethnicity**		
Maasai	1	1
Non-Maasai	1.7 (1.1–2.7)^b^	1.6 (1.1–2.3)^b^
**Wealth index**		
Poorest	1	1
Least poor	2.0 (1.4–3.0)^b^	2.0 (1.2–3.3)^b^
**Oral hygiene status**		
Poor		1
Good		0.7 (0.5–0.9)^b^
**DMFT**		
DMFT = 0		1
DMFT > 0		3.1 (2.1–4.5)^b^
**Dental fluorosis**		
TF 0–4		1
TF 5–9		1.5 (0.9–2.4)
**TMD pain**		
2Q/TMD^a^ = 0		1
2Q/TMD > 0		3.9 (2.4–6.2)^b^

Step 1: model fit Nagelkerke’s *R*^2^ = 0.103, Model Chi-Square = 55.407, df = 4, *p* < 0.001

Step 2: model fit Nagelkerke’s *R*^2^ = 0.199, model Chi-square = 110.178, df = 8, *p* < 0.001

^a^2Q/TMD Two epidemiological questions regarding TMD pain

^b^Statistically significant

Statistically significant effects of two-way interactions, occurred for ethnicity x age (OR = 4.4, 95% CI 1.7–11.3), ethnicity x dental caries (OR = 4.6, 95% CI 2.1–10.0) and ethnicity x self-reported TMD pain (OR = 8.0, 95% CI 3.6–17.8) (Table 4). As shown in Table 5, stratified logistic regression analysis by ethnic group revealed that among non-Maasais only, older adolescents were more likely than their younger aged counterparts to report at least one OIDP (OR = 3.7, 95% CI 1.1–12.8). Adolescents with (dental caries) DMFT> 0 were 2.8 (95% CI 1.4–5.5) and 3.3 (95% CI 1.2–9.0) times more likely to report oral impacts in the Maasai and non-Maasai groups, respectively. Maasai and non-Maasai adolescents with self-reported TMD pain were more likely to report oral impacts than their counterparts with no self-reported TMD pain. The corresponding OR and 95% CI were 3.0 (1.7–5.2) and 9.0 (3.3–25.0), respectively.

**Table 4:** Statistically significant effects of two-way interactions between adolescents’ social and clinical characteristics and OIDP (cluster adjusted), odds ratios (OR) ad 95% confidence interval (CI)

Variable	Step 2 OR (95% CI)
**Ethnicity x age**	
Maasai × 12–14 years	1
Non Maasai × 15–17 years	4.4 (1.7–11.3)^b^
**Ethnicity x DMFT**	
Maasai x DMFT = 0	1
Non Maasai x DMFT> 0	4.6 (2.1–10.0)^b^
**Ethnicity x TMD pain**	
Maasai x 2Q/TMD^a^ = 0	1
Non-Maasai x 2Q/TMD > 0	8.0 (3.6–17.8)^b^

^a^2Q/TMD Two epidemiological questions regarding TMD pain

^b^Statistically significant

**Table 5:** Logistic regression for the association between adolescents’ social and clinical characteristics and OIDP, according to ethnic belongingness (cluster adjusted). Odds ratios (OR) ad 95% confidence interval (CI)

Variable	Maasai		Non-Maasai	
	Step 1 OR (95% CI)	Step 2 OR (95% CI)	Step 1 OR (95% CI)	Step 2 OR (95% CI)
**District of residence**				
Monduli	1	1	1	1
Longido	0.2 (0.1–0.4)^b^	0.4 (0.2–0.7)^b^	0.7 (0.3–1.7)	0.8 (0.3–2.5)
**Age**				
12–14 years	1	1	1	1
15–17 years	1.1 (0.6–2.0)	0.9 (0.5–1.7)	3.1 (1.0–9.1)	3.7 (1.1–12.8)^b^
**Wealth index**				
Poorest	1	1	1	1
Least poor	2.0 (1.3–3.0)^b^	1.9 (1.2–2.9)^b^	1.4 (0.4–4.5)	1.7 (0.5–6.3)
**Oral hygiene status**				
Poor		1		1
Good		0.6 (0.3–1.0)		0.6 (0.3–1.5)
**DMFT**				
DMFT = 0		1		1
DMFT > 0		2.8 (1.4–5.5)^b^		3.3 (1.2–9.0)^b^
**Dental fluorosis**				
TF 0–4		1		1
TF 5–9		1.7 (1.0–2.9)		1.0 (0.4–2.7)
**TMD pain**				
2Q/TMD^a^ = 0		1		1
2Q/TMD > 0		3.0 (1.7–5.2)^b^		9.0 (3.3–25.0)^b^

^a^2Q/TMD Two epidemiological questions regarding TMD pain

^b^Statistically significant

## Discussion

This study is among the first to estimate the prevalence-and socio-demographic- and clinical covariates of oral impacts on daily performances among adolescents living in Maasai population areas of the Arusha region in Tanzania. The prevalence of OIDP was moderate and amounted to about 15 and 20% among the Maasai and non-Maasai ethnic groups, respectively. Independent of ethnic group belongingness, adolescents who were the least poor according to the wealth index utilized and those who presented with oral diseases were most likely to report oral impacts. The present findings revealed significant differences by ethnic groups in the relationships between socio-demographic- and clinical covariates and oral impacts. Older adolescents were more likely than their younger counterparts to report oral impacts among the non-Maasais only. Dental caries experience and self-reported TMD pain was significantly associated with oral impacts among both ethnic groups but more strongly among the non-Maasais than among their Maasai counterparts.

In this study, about 16% reported at least one oral impact on daily performance during the past 3 months (15% among Maasais and 20% among non-Maasais). These prevalence rates are lower than those reported previously among adolescents of similar ages in sub Saharan Africa ranging from 29 to 62% [4–6, 36]. Higher prevalence of OIDP has also been reported among the adolescents from other countries, for example Brazil (37%), Italy (67%), China (46%) and Thailand (85%) [37–40]. The relatively low prevalence of oral impacts among Maasai- and non-Maasai adolescesnts is most likely related to the low occurrence of oral diseases generally and dental caries particularly observed in those populations. In accordance with some previous reports from sub Saharan Africa, difficulties with eating food and cleaning teeth were the most frequent oral impacts affecting adolescents in this study [4–6].

The present differences in OIDP across sociodemographic- and clinical variables confirm the social and clinical gradients observed in oral health of adolescent/adult populations worldwide [41, 42]. Although Longido district was most populated with non-Maasai adolescents, Longido residents were less likely to report oral impacts than their counterparts in Monduli district. This difference might be attributed to variation in prevalence and severity of oral diseases as well as differences in the socio-demographic distribution between the two districts. Non-Maasais had higher odds of reporting any OIDP than the Maasais and ethnic group was strongly and independently associated with oral impacts after adjustment for oral diseases and socio-demographic factors. Although comparable studies from sub Saharan Africa are lacking, reports from high income countries have shown that non-White individuals or minority ethnic groups are more likely to have oral impacts than their white majority ethnic group counterparts [43, 44]. The influence of ethnicity on oral health is linked to, socioeconomic, behavioral and psychosocial factors that varied across the ethnic groups [45]. In this study, most adolescents were from poor families and lived in rural remote areas where social services are limited. All these exposed them to various risk factors of oral diseases and affected their psychological, social and quality aspects of their life^..^ Moreover, the present findings and also previous ones have shown that non-Maasai adolescents are more frequently affected by oral diseases than their Maasai counterparts [23]. Those from the least poor families were more likely to report oral impacts than those from the poorest families. This is contrary to other studies reporting that the poorest families report most oral impacts [4, 46]. In this study, adolescents from the least poor families might have the easiest access to sugary foods and thus being those most exposed to development of dental caries. In accordance with the present findings, a previous study reported that Tanzanian adolescents from least poor families, according to the family wealth index, reported dental pain and other oral problems more frequently than their counterparts from the poorest families [5].

In accordance with previous studies but contrary to others, the present one demonstrated a strong and independent association between indicators of oral health status, namely dental caries and self-reported TMD pain, and oral impacts [6, 27, 47–49] (Table 3). Moreover, adolescents with good oral hygiene had lower odds than those with poor oral hygiene to report oral impacts. This finding is contrary to other studies from Tanzania which found no significant differences in oral impacts according to level of oral hygiene status [4, 6]. Poor oral hygiene is an outcome of irregular tooth cleaning and may lead to gingivitis and increase the risk for periodontitis [50], which has a negative impact on oral health related quality of life [51, 52]. Dental caries, if left untreated, can affect adolescent’s quality of life through dental pain leading to deterioration in oral functioning, emotional state as well as social roles [53]. The positive association observed between self-reported TMD pain and oral impacts supports findings from previous studies [54–56]. The similarity in these findings indicates that self-reported TMD pain affects OHRQoL across various populations. The OIDP index measures only ultimate impacts and statistically significant relationships can be difficult to demonstrate when the prevalence of one of the covariates is low. Thus, it is noteworthy that the observed associations between clinical indicators and oral impacts were statistically significant despite a relatively low prevalence of oral impacts in the present study population. The prevalence of tooth wear/dental erosion in this population was relatively low and did not associate with oral impacts on daily performance. Similar negative findings between oral health related quality of life indicators and low prevalence of tooth wear have been reported elsewhere [57]. On the other hand, if prevalence and severity of tooth wear increases in the future caused by changes in behavioral pattern and/or socioeconomic conditions, it could result in an impact on oral health related quality of life as reported by others [58]. In the studied society, it is likely that today’s traditional lifestyle and relatively low prevalence oral diseases will be subjected to a change into a more modern way of living in the future. In this regard, harmful choices of behavior may arise which may increase the risk for development oral diseases. This perspective has to be considered in future public dental health planning.

### Study limitations

The present study used a large sample size providing more reliable results with greater precision and power. In addition, the study provided a detailed oral clinical examination and used an oral quality of life inventory previously validated for use in Tanzanian and other non-occidental context. Although schools were selected randomly and in spite of a high response rate, the possibility of selection bias cannot be overlooked. The rigorous selection criteria utilized might have resulted in a recruited study population that was not strictly representative of the general adolescent population in the study area. The structured interviewer-administered schedule used to assess self-reported data might be subject to social desirability, acquiescence, and recall biases. Attempts were made to minimize these biases by informing the participants that their responses were confidential and that no-one could link their names to their responses. Furthermore, the cross-sectional design utilized makes it difficult to establish causal relationships. In addition, our plan was to invite all adolescents aged 12–14 years. However, during interview we invited all available adolescents without separating them according to age. During analysis we realized that the age range was between 11 to 26 years. So to reduce this wide age range we decided to exclude those below 12 years and above 17 years.

## Conclusion

The prevalence of oral impacts was moderate but higher among the non-Maasai-than Maasai- adolescents, attending rural primary schools in the Maasai population areas of Tanzania. This study also confirmed socioeconomic and oral clinical disparities in OIDP, some of which differed according to ethnicity. Caries experience and self-reported TMD pain associated more strongly with OIDP among the non-Maasais than among the Maasais. The results are important for public oral health decision makers who plan strategies for optimal oral health and quality of life among adolescents belonging to minority groups in Tanzania. This is important for the prevention of oral diseases and improvement of oral health related quality of life among the adolescents of the under privileged ethnic groups.

## Data Availability

The datasets used and/or analysed during the current study are available from the corresponding author on request.

## Abbreviations

2Q/TMD: Two epidemiological Questions regarding Temporomandibular Disorder pain
2Q/TMD > 0: A positive response to any or both of the two epidemiological questions regarding Temporomandibular Disorder pain
2Q/TMD = 0: A negative response to both epidemiological questions regarding Temporomandibular Disorder pain
CI: Confidence interval
C-OIDP: Child-oral impact on daily performance
CI-S: Simplified calculus index
D: Design effect
DI-S: Simplified Debris Index
DMFT: Decayed Missing Filled Tooth
GBI: Gingival Bleeding Index
OHI-S: Simplified Oral Hygiene Index
OHRQoL: Oral health related quality of life
OIDP: Oral impact on daily performance
OIDPSC: Oral Impact on Daily Performance Simple Count score
OR: Odds Ratio
PCA: Principal Component Analysis
SD: Standard deviation
SPSS: Statistical Package for Social Sciences
TF-index: Thylstrup-Fejerskov-index
TMD: Temporomandibular disorder
WHO: World Health Organisation
